# 58例小细胞癌与鳞癌复合型肺癌的临床分析

**DOI:** 10.3779/j.issn.1009-3419.2016.10.04

**Published:** 2016-10-20

**Authors:** 扬 罗, 玉 门, 周光 惠, 峻岭 李, 学志 郝, 镨元 邢

**Affiliations:** 1 100021 北京，国家癌症中心/中国医学科学院北京协和医学院肿瘤医院内科 Department of Medical Oncology, National Cancer Center/Cancer Hospital, Chinese Academy of Medical Sciences and Peking Union Medical College, Beijing 100021, China; 2 100021 北京，国家癌症中心/中国医学科学院北京协和医学院肿瘤医院放射治疗科 Department of Radiation Oncology, National Cancer Center/Cancer Hospital, Chinese Academy of Medical Sciences and Peking Union Medical College, Beijing 100021, China

**Keywords:** 肺肿瘤, 小细胞癌, 鳞癌, 复合型癌, Lung neosplasms, Small cell carcinoma, Squamous cell carcinoma, Combined carcinoma

## Abstract

**背景与目的:**

小细胞癌与鳞癌复合型肺癌少见，本研究分析其临床病理特征和治疗现状，探讨影响预后的因素。

**方法:**

回顾性分析2004年1月-2012年12月58例病理细胞学证实的小细胞癌与鳞癌复合型肺癌患者的资料，采用*Kaplan*-*Meier*法计算生存率，*Log*-*rank*法进行单因素预后分析，*Cox*风险回归模型分析影响总生存（overall survival, OS）的因素。

**结果:**

全组患者的OS为0.3个月-124.3个月，中位OS为22.7个月。单因素分析显示：初诊卡氏评分＜80分、广泛期、肿瘤-淋巴结-转移（tumor-node-metastasis, TNM）分期晚是影响OS的不良预后因素（*P*＜0.05）。多因素分析显示，只有TNM分期是独立的影响OS的因素（*P*=0.019）。治疗多采取化疗为主的综合模式治疗，远处转移仍是治疗失败的主要原因。

**结论:**

小细胞癌与鳞癌复合型肺癌患者的治疗多采用以化疗为主的综合治疗模式，TNM分期是独立的预后影响因素。

## Abstract (en)

**Background and objective:**

Small cell lung cancer combined with squamous cell carcinoma are rare. The aim of this study was to analyze the clinicopathological characteristics and treatment, and explored the prognostic factors of this disease.

**Methods:**

Between January 2004 and December 2012, 58 patients with cytopathologically confirmed small cell lung cancers combined with squamous cell carcinoma were retrospectively analyzed. *Kaplan*-*Meier* methods were used to calculate the survival rate, and *Log*-*rank* test was used to examine differences between arms. The *Cox* regression model was used to analyze the independent factors affecting the overall survival (OS).

**Results:**

The OS of the 58 patients was 22.7 months with a range of 0.3 to 124.3 months. In univariate analysis, Karnofsky performance score before treatment, extensive disease, tumor stage were the considered prognostic factors affecting the OS rate (*P* < 0.05). *Cox* multivariate analysis showed that only the tumor-node-metastasis (TNM) stage was the independent prognostic factor (*P*=0.019). The majority of the patients received multimodality therapy and chemotherapy was the main treatment. Distant metastasis was the main reasonfor the treatment failure.

**Conclusion:**

Combined therapy with chemotherapy as the main treatment should be adopted in therapeutic regimen of the patients with small cell lung cancers combined with squamous cell carcinoma. TNM stage was the independent prognostic factor influencing the OS.

复合型小细胞肺癌（combined small cell lung cancer, C-SCLC）是一类由SCLC与非小细胞肺癌（non-SCLC, NSCLC）成分相混合的癌，其中NSCLC成分可以是鳞癌、腺癌、大细胞癌，甚至是少见的梭形细胞癌、巨细胞癌、肉瘤样癌等，且混合的NSCLC病理成分可以为一种或多种，其中以混合鳞癌最为常见^[1-3]^。SCLC和肺鳞癌均好发于老年吸烟男性，中央型多见，但SCLC更易于转移，对化放疗更为敏感，而鳞癌以手术治疗为主。既往尚无专门针对SCLC与鳞癌复合型肺癌的相关报道。因此，我们开展了回顾性研究，结合文献探讨SCLC与鳞癌复合型肺癌的临床病理特征，治疗和预后。

## 1 资料与方法

### 1.1 一般临床资料

2004年1月-2012年12月在中国医学科学院肿瘤医院治疗的SCLC和鳞癌复合型肺癌患者共58例，患者的临床资料详见表 1，全部诊断均经组织病理学证实，包括原发灶手术切除标本32例、支气管镜活检和刷检细胞学16例、肺穿刺活检2例、转移淋巴结活检3例、其他转移部位活检1例、痰细胞学和支气管镜活检共同诊断1例、痰细胞学和肺穿刺活检共同诊断1例，支气管镜活检和肺穿刺病理共同诊断1例、支气管镜活检和转移淋巴结穿刺共同诊断1例。分期检查包括体格检查、胸部计算机断层扫描、正电子发射计算机断层显像、颈腹部超声、脑磁共振成像和骨扫描等，采用2009年美国癌症联合委员会（American Joint Committee on Cancer, AJCC）第7版肿瘤-淋巴结-转移（tumor-node-metastasis, TNM）分期及美国退伍军人肺癌协会（Veterans Administration Lung Study Group, VALSG）分期^[4]^（局限期指肿瘤位于一侧胸腔，即使发生了局部转移和同侧锁骨上淋巴结转移，只要他们与原发灶能覆盖于同一放射野，也属于局限期，没有胸腔外转移也被称为局限期，除此之外属于广泛期）。

**1:** 患者的一般临床资料 The characteristics of the patients

Characteristics	*n*	Proportion (%)
Age (yr)		
≤60	34	58.6
＞60	24	41.4
Gender		
Male	48	82.8
Female	10	17.2
Smoking history		
Yes	46	79.3
No	12	20.7
Karnofsky performance score		
≥80	50	86.2
＜80	8	13.8
Weight loss		
Yes	7	12.1
No	51	87.9
X-ray typing		
Central	52	89.7
Peripheral	6	10.3
Location		
Left lung	29	50.0
Right lung	29	50.0
Upper lobe	33	56.9
Middle lobe	4	6.9
Lower lobe	21	36.2
VALSG staging		
Limited disease	45	77.6
Extensive disease	13	22.4
AJCC 7^th^ stage		
Ⅰ	4	6.9
Ⅱ	11	19.0
Ⅲ	31	53.4
Ⅳ	12	20.7
Component		
Mostly in SCLC	50	86.2
Mostly in sqCLC	8	13.8
SCLC: small cell lung cancer; sqCLC: squamous cell lung cancer; AJCC: American Joint Committee on Cancer; VALSG: Veterans Administration Lung Study Group.		

### 1.2 研究因素

包括患者的一般状况（年龄、性别、吸烟史、卡氏评分、体重是否减轻）；肿瘤相关情况（肿瘤大小、肿瘤位置、TNM分期、VALSG分期、鳞癌成分所占比例）；治疗相关情况。另外，按照分期对患者的治疗和失败模式进行分析。不吸烟定义为吸烟＜100支，体重减轻定义为半年内体重下降＞5%。

### 1.3 统计学方法

随访截至2015年12月，4例患者失访，随访率为93.1%。总生存（overall survival, OS）定义为从首次治疗开始至死亡或末次随访的时间。采用SPSS 20.0软件行*Kaplan*-*Meier*法生存分析，*Log*-*rank*法单因素预后分析，*Cox*风险回归模型进行多因素分析。*P*＜0.05为差异有统计学意义。

## 2 结果

### 2.1 临床病理特点

全组患者的中位年龄为58岁（29岁-79岁），男性较女性多见，分别为48例和10例，比例约为4.8:1。大部分患者（46例，79.3%）有吸烟史。多数患者一般状况较好，50例（86.2%）初诊时卡氏评分≥80分，仅有7例（12.7%）体重减轻者。小细胞癌和鳞癌复合型肺癌以中央型居多（52例，89.7%）；局限期（45例）较广泛期多见（3.5:1）；采用AJCC第7版分期，Ⅰ期、Ⅱ期、Ⅲ期和Ⅳ期的患者分别为4例（6.9%）、11例（19.0%）、31例（53.4%）和12例（20.7%）；多数病例混合成分以小细胞癌为主，少数（8例，13.8%）鳞癌的比例超过50%。

### 2.2 生存结果

随访截至2015年12月，失访4例，死亡33例（包括4例非肿瘤相关死亡，分别为1例术后感染未控制、1例脑血栓、1例肺部感染和1例房颤），1年、3年、5年生存率分别为81.9%、51.4%和42.1%。全组的OS为0.3个月-124.3个月，中位OS为22.7个月。

### 2.3 预后分析

单因素分析显示、初诊卡氏评分＜80分、广泛期、TNM分期晚是OS的不良预后因素（*P*＜0.05），而男性、年龄、吸烟史、体重减轻、肿瘤X线分型、肿瘤所在的位置、混合成分中小细胞癌和鳞癌所占比例等因素未显示与预后相关（*P*＞0.05）。将*P*＜0.05的3个变量进行*Cox*回归分析（表 2），只有TNM分期是独立的预后影响因素（*P*=0.019）。Ⅰ期、Ⅱ期、Ⅲ期、Ⅳ期患者的5年OS率分别为100.0%、61.4%、38.5%和12.5%（χ^2^=11.637，*P*=0.009，图 1）。

**2:** 预后因素的*Cox*回归分析 Multivariate prognostic analysis by *Cox* model

Factors	B	df	*P*	Exp(B)	95%CI for Exp(B)	
					Lower	Upper
Tumor-node-metastasis (TNM) staging	0.980	1	0.019	2.665	1.176	6.041
VALSG staging	-0.731	1	0.244	0.481	0.141	1.647
Karnofsky performance score	0.947	1	0.054	2.579	0.985	6.751

**1:**
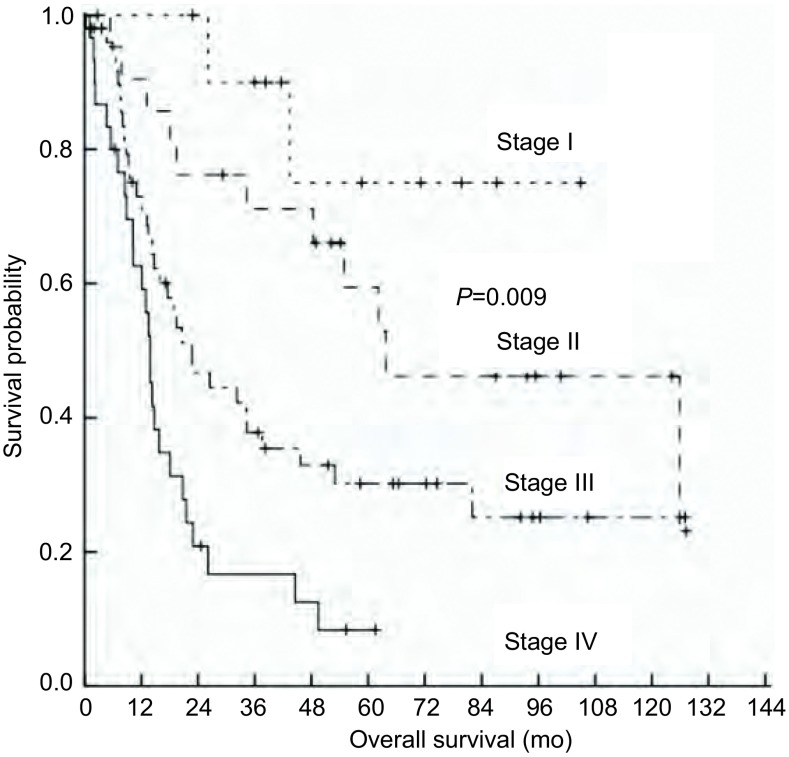
Ⅰ期-Ⅳ期患者的生存曲线 Survival curve of the patients with stage Ⅰ-Ⅳ

### 2.4 各期患者的治疗和失败模式

各期患者的治疗模式见表 3，可以看出小细胞癌复合鳞癌的患者的治疗模式以综合治疗为主。化疗在各个期别的治疗中均占有主要的作用，对于Ⅰ期和Ⅱ期病例，局部治疗以手术为主，Ⅲ期患者的局部治疗手术和放疗并重，Ⅳ期患者以化疗为主。失败模式详见表 4，本组共有4例患者失随，12例患者随访到因肿瘤死亡，具体不详。小细胞癌复合鳞癌的患者的治疗失败仍以远地转移为主，常见转移部位依次为脑、骨、肺、肝脏、区域外淋巴结及肾上腺等。

**3:** Ⅰ期-Ⅳ期患者的治疗模式 The treatment modalities in the patients with stage Ⅰ-Ⅳ

Item	Stage Ⅰ (*n*=4)	StageⅡ (*n*=11)	Stage Ⅲ (*n*=31)	Stage Ⅳ (*n*=12)
Treatment methods				
S	4 (100%)	9 (81.8%)	18 (58.1%)	3 (25.0%)
C	3 (75.0%)	9 (81.8%)	26 (83.9%)	9 (75.0%)
R	0 (0)	4 (36.4%)	17 (54.8%)	8 (66.3%)
Treatment modes				
Single-mode	1 (25.0%)	3 (27.3%)	9 (29.0%)	4 (33.3%)
S+C	3 (75.0%）	4 (36.4%)	5 (16.1%)	1 (8.3%)
S+R	0 (0)	0 (0)	0 (0)	1 (8.3%)
C+R	0 (0)	1 (9.1%)	9 (29.0%)	6 (50.0%)
S+C+R	0 (0)	3 (27.3%)	8 (25.8%)	0 (0)
S: surgery; C: chemotherapy; R: radiotherapy.				

**4:** Ⅰ期-Ⅳ期患者的首次治疗失败 Site of first recurrence of the patients with stage Ⅰ-Ⅳ

Event type	Stage Ⅰ (*n*=4)	StageⅡ (*n*=11)	Stage Ⅲ (*n*=31)	Stage Ⅳ (*n*=12)
No. of total recurrences				
Local only	0	0	1	0
Regional nodal	0	1	2	0
Local and distant	0	2	9	3
Distant only	0	1	2	3
No. of additional events				
Death	0	2	5	5
Lost of follow-up	0	0	3	1

## 3 讨论

C-SCLC是SCLC中的一类少见的特殊病理亚型，包含了SCLC和NSCLC两种不同的癌成分，既往报道其发病率仅占全部SCLC的1%-3%^[1, 5]^，关于C-SCLC的组织起源存在争议，尚未确定C-SCLC中的不同组织成分是由同一个肿瘤干细胞在增殖的过程中沿着不同的分化方向不断分化而形成，还是发生于肺的两种不同组织类型的恶性肿瘤结合而形成。多数学者推测SCLC来源于神经脊细胞，而神经脊细胞已经被证实具有多向分化的潜能，因此这种双向分化的肿瘤很可能来源于同一个肿瘤干细胞，在不同的微环境中可以分化为神经内分泌细胞或是鳞状细胞^[6]^。Wagner等^[7]^通过免疫组化及DNA微阵列杂合性缺失分析发现，C-SCLC中两种不同成分具有极相似的免疫表型和基因表型，进一步证实NSCLC成分是由SCLC成分转变而来。

C-SCLC以SCLC复合鳞癌最为多见，多数报道为50%左右，最高的报道可达90%^[8]^，这可能是因为鳞癌和SCLC的发病均与吸烟相关，并且两者的组织发生部位一致，均来源于鳞状上皮基底细胞层，在组织发生上具有一定的相关性。另外采用充分的肿瘤组织进行病理学检查是保证C-SCLC不被漏诊的前提，而SCLC和鳞癌均多发于段支气管以上，痰细胞学和纤支镜易取得标本，本组中26例未接受手术的患者中，20例患者由纤支镜活检或刷检以及痰细胞学诊断。

多数报道在C-SCLC中，SCLC复合鳞癌患者的预后优于其他混合病理成分，因此病理诊断时应对C-SCLC组织类型予以说明，高、中分化鳞状细胞癌，H & E染色标本就可以看到明显的鳞癌癌巢及角化现象，而对于低分化鳞癌或小的活检标本则需进一步鉴别诊断，免疫组织化学在鉴别诊断中具有重要作用，甲状腺转化因子1（thyroid transcription factor-1, TTF-1）在大多数SCLC、肺腺癌、小部分大细胞未分化癌和不典型类癌、少数典型类癌中阳性表达，而肺鳞癌中不表达。P63表达于低分化鳞癌，而在SCLC中不表达。因此，TTF-1、P63再联合使用传统的细胞角蛋白（cytokeratin, CK）高、CK低可以用于鉴别其复合成分是否为低分化鳞癌^[9]^。

本组患者多为老年吸烟男性，以中央型多见，与单纯型SCLC相似，但本组中局限期病例占77.8%，其中早期（Ⅰ期-Ⅱ期）病例占全组的25.9%，则远远高于单纯型SCLC文献^[10]^报道的只有20%-25%的患者属局限期，仅10%的患者为早期（Ⅰ期-Ⅱ期）。其原因可能是本组大部分病例经手术病理确诊，而手术患者多为早期病变，另外很可能与鳞癌成分相关，与SCLC相比，鳞癌更容易局部生长，而发生淋巴和血行转移较晚。

尚无有关SCLC复合鳞癌患者预后的报道，本组总的中位OS为22.7个月，单因素分析显示、初诊卡氏评分＜80分、广泛期、TNM分期晚是OS的不良预后因素，而*Cox*回归分析显示只有TNM分期是独立的预后影响因素（*P*=0.019）。Ⅰ期、Ⅱ期、Ⅲ期、Ⅳ期患者的5年OS率分别为100.0%、61.4%、38.5%和12.5%（χ^2^=11.637, *P*=0.009）。

针对C-SCLC治疗的文献报道很少，其治疗原则主要借鉴SCLC的治疗模式，强调综合治疗，但综合治疗模式仍不统一。对于纯SCLC患者而言，即使是局限期的患者，标准治疗仍为同步放化疗，手术仅限于小的、淋巴结阴性的肿瘤非常局限的患者。这主要是因为单纯型SCLC对化疗相当敏感，有效率高达60%-80%^[11]^，虽然临床上，C-SCLC基本采用与单纯型SCLC相同的化疗方案，但是其对化疗的反应较单纯型SCLC差，有效率约为50%左右，导致C-SCLC对化疗疗效下降的原因可能是由于C-SCLC中混合的NSCLC成分对SCLC常规方案的敏感性较差。对化疗敏感性的下降可能导致局部治疗对于C-SCLC的治疗非常重要，Hage等^[12]^研究结果显示，对于高度选择（外周型或临床分期为Ⅰ期）的C-SCLC患者，手术因其相对较高的治愈率而具有非常重要的价值，Ⅰ期C-SCLC患者手术后的5年生存率为31%，其中T1N0M0和T2N0M0患者的5年生存率分别为50%和25%。而对于临床分期为Ⅲ期的患者不建议手术，因为这部分患者不能从手术中获益，新辅助化疗和/或辅助化疗的价值仍无定论，但通常被认为可以减少远处转移的发生。而对于局部晚期的患者，门玉等^[13]^报道放疗可以明显提高局部晚期（Ⅲa期、Ⅲb期）（*P*=0.032）、淋巴结阳性（*P*=0.006）或术后阳性淋巴结＞4个（*P*=0.025）患者的OS。本组患者的治疗模式仍采用以化疗为主的综合治疗模式。对于Ⅰ期和Ⅱ期病例，局部治疗以手术为主，Ⅲ期患者的局部治疗手术和放疗并重，Ⅳ期患者以化疗为主。治疗失败以远地转移为主，也说明化疗的疗效亟待提高。

C-SCLC由于发病率低，多数文献将其合并讨论，但是由于混合成分的不同，其临床特点也各有不同，因此，本文单独讨论SCLC复合鳞癌的病例，试图进一步明确其临床特征，研究结果显示TNM分期是SCLC复合鳞癌患者的独立的预后影响因素，对于可以手术的早期病例，化疗结合手术的疗效突出，期待以后有更大规模的研究进一步验证本研究结果。
